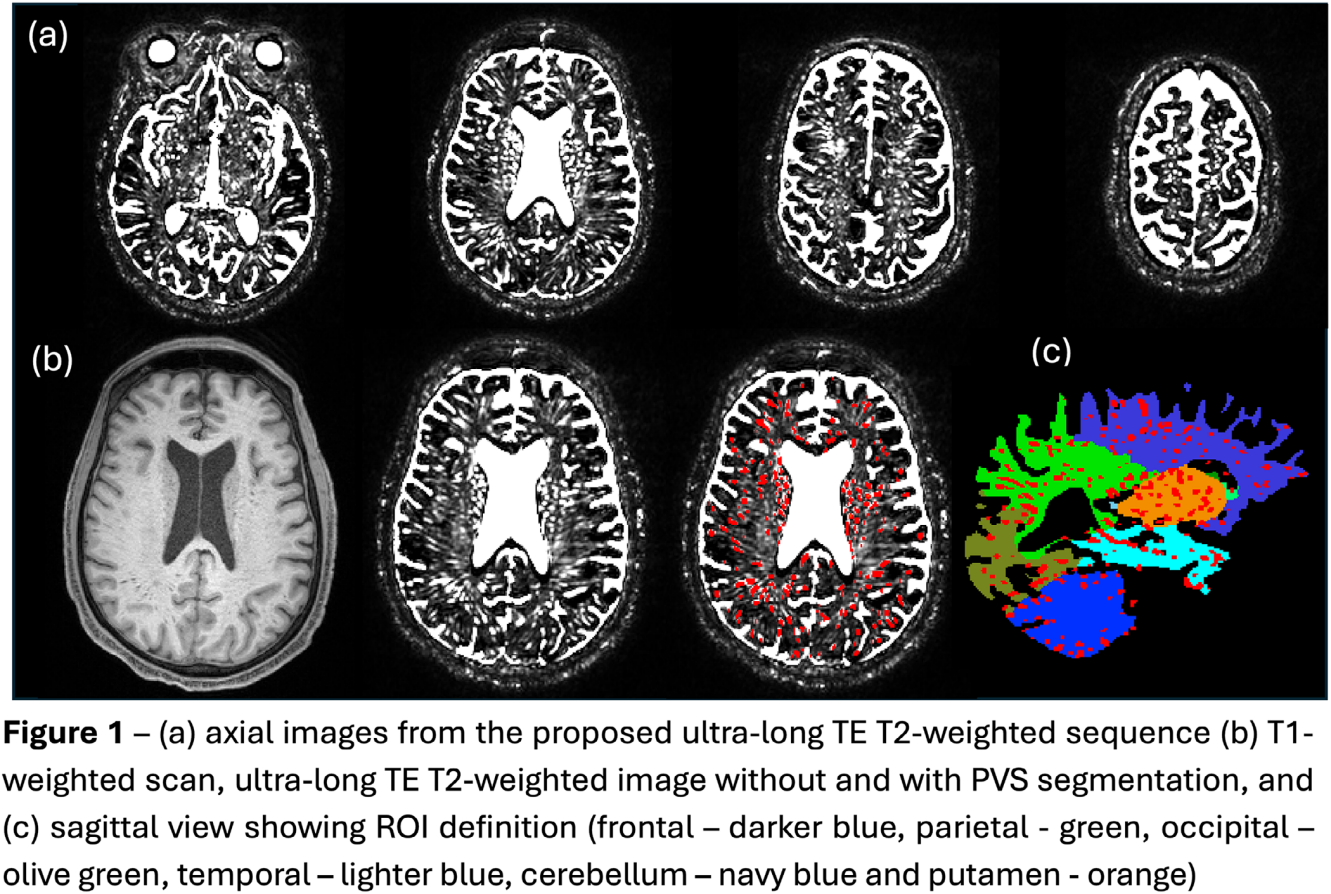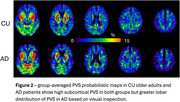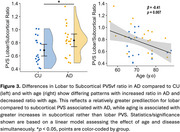# Segmentation and Quantification of Perivascular Spaces in Early Alzheimer’s Disease using Ultra‐long TE T2‐weighted MRI

**DOI:** 10.1002/alz70861_108886

**Published:** 2025-12-23

**Authors:** Manuel Taso, Christopher A Brown, Sandhitsu R. Das, Long Xie, Emily McGrew, Danielle Hing, Valerie Humphreys, Farraday N Johnson, Laura Schankel, Paul A. Yushkevich, Ilya M. Nasrallah, Dawn Mechanic‐Hamilton, John A. Detre, David A. Wolk

**Affiliations:** ^1^ Siemens Medical Solutions, Malvern, PA USA; ^2^ University of Pennsylvania, Philadelphia, PA USA; ^3^ Siemens Healthineers, Princeton, NJ USA

## Abstract

**Background:**

Perivascular spaces (PVS) are small fluid‐filled structures that are of major interest in both cerebral small vessel disease (cSVD) and neurodegeneration, particularly in the pathophysiology of AD and cerebral amyloid angiopathy (CAA). Current approaches to measure and quantify PVS rely on multi‐contrast combination or high‐field MRI to enhance visualization. We propose here an optimized imaging sequence for selective imaging of intracranial fluids with high resolution and clinically practical scan time capable of detecting AD‐related PVS enlargement.

**Method:**

19 patients with MCI or mild Dementia due to AD scanned prior to initiation of anti‐amyloid therapy (73±7yo) and 19 cognitively unimpaired (CU) older adults (71±7yo) were enrolled and scanned on a Siemens Prisma 3T MRI at the University of Pennsylvania. We acquired a 3D ultra‐long‐TE T2‐weighted sequence (ulTE‐T2, TR/TE=5000/876ms, FA=75deg, BW=681Hz/pix, 1mm isotropic, GRAPPA=4) in 3min 40sec. Motion‐corrupted scans (4 AD, 2 CU) were excluded. Images were co‐registered with a T1‐weighted volume and warped into MNI space. A 3D‐Frangi filter was used to automatically segment PVS within WM‐masked regions‐of‐interest (ROI). PVS volume fractions (PVS_vf_ = V_pvs_/V_ROI_) were computed in various lobal (parietal/occipital/frontal/temporal) and subcortical regions of interests and whole brain PVS probability maps were also produced by averaging individual PVS segmentations. A lobar/subcortical PVS_vf_ ratio was calculated and then compared between AD and CU.

**Result:**

Figure 1 shows the sensitivity towards PVS of the ulTE‐T2 sequence and automatic segmentation results. Group probability maps show high PVSvf in the basal ganglia and in posterior > anterior WM (Figure 2). PVSvf lobar/subcortical ratio was higher in AD compared to CU (Figure 3, *β*=0.68 [0.11,1.26], *t*(35)=2.42, *p*=0.021) after controlling for age, which was negatively associated with PVSvf ratio (*β*=‐0.41 [‐0.70, ‐0.12], *t*(35)=‐2.84, *p*=0.007).

**Conclusion:**

Fluid‐specific PVS imaging can be performed within clinically feasible scan times. Early results show strong visual and quantitative sensitivity towards PVS. Furthermore, individuals with AD show increased lobar to subcortical PVS volume ratio compared to CU both on visual assessment and with quantitative evaluation. Ongoing future directions include implementing variant sequences with improved motion robustness and longitudinal follow‐up to evaluate the effects of anti‐amyloid therapy on PVS.